# Antigen specific activation of cytotoxic CD8^+^ T cells by *Staphylococcus aureus* infected dendritic cells

**DOI:** 10.3389/fcimb.2023.1245299

**Published:** 2023-10-26

**Authors:** Adèle Friot, Sophia Djebali, Séverine Valsesia, Peggy Parroche, Maxence Dubois, Jessica Baude, François Vandenesch, Jacqueline Marvel, Yann Leverrier

**Affiliations:** Universite Claude Bernard Lyon 1, Centre International de Recherche en Infectiologie, Inserm U1111, CNRS UMR5308, École Normale Supérieure de Lyon, Lyon, France

**Keywords:** *Staphylococcus aureus*, antigen-specific activation, cytotoxic CD8^+^ T cells, DC2.4, dendritic cells, cytotoxicity, lymphocytes

## Abstract

*Staphylococcus aureus* (*S. aureus*) is a pathogen associated with a wide variety of diseases, from minor to life-threatening infections. Antibiotic-resistant strains have emerged, leading to increasing concern about the control of *S. aureus* infections. The development of vaccines may be one way to overcome these resistant strains. However, *S. aureus* ability to internalize into cells – and thus to form a reservoir escaping humoral immunity – is a challenge for vaccine development. A role of T cells in the elimination of persistent *S. aureus* has been established in mice but it remains to be established if CD8^+^ T cells could display a cytotoxic activity against *S. aureus* infected cells. We examined *in vitro* the ability of CD8^+^ T cells to recognize and kill dendritic cells infected with *S. aureus.* We first evidenced that both primary mouse dendritic cells and DC2.4 cell line can be infected with *S. aureus*. We then generated a strain of *S. aureus* expressing a model CD8 epitope and transgenic F5 CD8^+^ T cells recognizing this model epitope were used as reporter T cells. In response to *S. aureus*-infected dendritic cells, F5 CD8^+^ T cells produced IFN-γ in an antigen-specific manner and displayed an increased ability to kill infected cells. Altogether, these results demonstrate that cells infected by *S. aureus* display bacteria-derived epitopes at their surface that are recognized by CD8^+^ T cells. This paves the way for the development of CD8^+^ T cell-based therapies against *S. aureus*.

## 1 Introduction

*Staphylococcus aureus* bacteria (*S. aureus*) colonize approximatively 30% of the human population, mostly as a nasal commensal (Foster, 2005; Uebele et al., 2017). It can also cause superficial and deep-seated infections after traumatic or iatrogenic effractions of the cutaneous-mucous barriers or on the occasion of acquired alterations of the immune defenses (Foster, 2005). Following the spread of multi-antibiotic resistant variants and the failure of several vaccination trials against this pathogen (Giersing et al., 2016), *S. aureus* infections have become a major public health problem. The primary defense against *S. aureus* infection is the innate immunity provided by neutrophils and infections can become more problematic to treat as *S. aureus* has the capacity to invade host cells, inducing chronic forms of infection and leading to long-term afflictions (Fraunholz and Sinha, 2012; Garcia-Moreno et al., 2022).

Although *S. aureus* wasn’t first described as an intracellular pathogen, this bacterium has the ability to enter into host cells, such as keratinocytes, osteoblasts, epithelial cells, myocytes, etc., through the interaction with host cell integrins (Fowler et al., 2000; Ahmed et al., 2001; Kintarak et al., 2004; Agerer et al., 2005; Abel et al., 2011; Darisipudi et al., 2018). Professional phagocytes, such as neutrophiles, dendritic cells (DCs) or macrophages, are also able to internalize *S. aureus* through phagocytosis (Kobayashi et al., 2010; Flannagan et al., 2016; Lacoma et al., 2017; Darisipudi et al., 2018). *S. aureus* manages to survive within eukaryotic host cells, being able to destroy the phagolysosome membrane and to escape into the cytoplasm. In the cytosol, *S. aureus* is able to replicate and to induce host cell death or to survive in a dormant state for extended periods of time as small colony variants (SCVs, Fraunholz and Sinha, 2012). *S. aureus* can also interfere with antigen processing and presentation on the Major Histocompatibility Complex (MHC) class II of the DCs, reducing their T cell-priming ability (Darisipudi et al., 2018).

The internalization of *S. aureus* by DCs is essential for immune responses against infections caused by this pathogen, as the depletion of these cells in mice impaired immunity against a bloodstream infection (Schindler et al., 2012). DCs are involved in the recruitment of neutrophils, and the co-culture of *S. aureus*-infected BDCA1^+^ DCs with T cells induces the proliferation of T cells and their production of IFN-γ (Jin et al., 2014). In a murine model, Zhang et al. demonstrated that after infection, the conventional DCs CD8α^-^ are the main producers of pro-inflammatory cytokines and activators of adaptive cells (Zhang et al., 2020). Plasmacytoid DCs are likewise involved in the immune response against *S. aureus* (Parcina et al., 2008; Parcina et al., 2013). Indeed, these cells are activated by *S. aureus* and, in response, produce pro-inflammatory as well as anti-inflammatory cytokines (Michea et al., 2013; Parcina et al., 2013). Hence, DCs may have an important role in immunity against *S. aureus* infection.

Although the innate and humoral immune responses against *S. aureus* have been the subject of much research (Krishna and Miller, 2012; Bröker et al., 2014; Dupont et al., 2018; Pelzek et al., 2018), the internalization and the persistence of *S. aureus* within host cells (immune and non-immune) allow the bacteria to escape from these responses (Dziewanowska et al., 1999; Ahmed et al., 2001; Abel et al., 2011; Josse et al., 2015; Surewaard et al., 2016). Thus, complete elimination of persistent *S. aureus* from infected organisms could require the intervention of cytotoxic cells, including CD8^+^ T cells. Cytotoxic CD8^+^ T cells kill target cells upon the recognition of their cognate epitope presented on the MHC class I (MHC-I) of infected cells or tumoral cells. In several murine models of infection (*Listeria monocytogenes*, *Mycobacterium tuberculosis or Salmonella typhi*), CD8^+^ T cells are involved in the clearance of intracellular bacteria (Kägi et al., 1994; Harty and Bevant, 1995; Sztein et al., 1995; Stenger et al., 1997; Stenger et al., 1998; Levine et al., 2001). A role of T cells in the elimination of persistent *S. aureus* has been established by Ziegler et al. using Rag2^-/-^ mice that exhibited a significantly improved capacity to control *S. aureus* when T cells were transferred (Ziegler et al., 2011). Moreover, in a model of cutaneous infection by *S. aureus* expressing an epitope from Ovalbumin, Egawa et al. have shown an antigen-specific activation of OT-I CD8^+^ T cells (Egawa et al., 2021). Although this activation was associated with a CD8^+^ T cell proliferation, it is not known if CD8^+^ T cells display a cytotoxic activity against *S. aureus* infected cells.

The propensity of *S. aureus* to internalize into host cells (Fraunholz and Sinha, 2012) explains the chronic forms of the infection and the failure to develop an effective vaccine, while opening new horizons in the development of treatments or preventive therapies based on the cytotoxic CD8^+^ T cell responses to intracellular *S. aureus*. The aim of the study was to examine the ability of CD8^+^ T cells to recognize and kill cells infected with *S. aureus*. To do so, we generated a *S. aureus* strain expressing a model epitope recognized by CD8^+^ T cells as well as GFP reporter protein to follow infection. We evidenced that DCs internalize *S. aureus* and present epitopes to antigen-specific CD8^+^ T cells. This recognition led to the production of IFN-γ and Interleukin-2 (IL-2) by CD8^+^ T cells and to an increased killing of infected cells.

## 2 Methods

### 2.1 Mice

Female C57BL/6 J were obtained from Charles River laboratories. F5 TCR transgenic mice, backcrossed on C57BL/6 background (Jubin et al., 2012), were bred and housed under specific pathogen-free conditions in the AniRA-PBES animal facility (Lyon, France). CD8^+^ T cells from these mice (called F5 CD8^+^ T cells hereafter) express a TCR that is specific for the ASNENMDAM epitope (called NP68 peptide hereafter) of influenza A nucleoprotein (aa 366-374). All experimental protocols were approved by the local ethic evaluation committee (CECCAPP, Lyon, France).

### 2.2 Bacterial strains and growth conditions

CD8^+^ T cell epitope (NP68) was inserted into LDH protein as its expression is maintained when *S. aureus* become chronic (Szafranska et al., 2014). Partial deletion of the *ldh* locus and in frame insertion of the NP68 motif in SH1000 strain of *S. aureus* was performed using pMAD (Arnaud et al., 2004). Chromosomal regions upstream and downstream of the *ldh* sequence were amplified by PCR (PCR1=nt 227938-229288, PCR2=nt 229289-230966 of *S.aureus* genome 8325); NP68 epitope (ASNENMDAM) and the four naturally NH2-flanking amino acid residues (GVQI) coding sequences were PCR synthetized. The NP68 coding DNA was cloned between the two *ldh* fragment onto pMAD, forming pLUG2143. This plasmid was electroporated in *E. coli*, then into RN4220 recipient strain and transferred to SH1000. Growth at non-permissive temperature (44°C) was followed by several subcultures at 30°C and 37°C to favor double crossing over as previously described (Arnaud et al., 2004), resulting in strain LUG2154. *gfp*-positive strains LUG2778 and LUG2779 were constructed by electroporating pCN38 into SH1000 and LUG2154, respectively.

LUG2778 and LUG2779 were inoculated into 4 ml of Brain Heart Infusion medium (37g.l^-1^ of Brain Heart Infusion Broth from Sigma-Aldrich) and grown overnight at 37°C, 5% CO_2_ and 200 rpm. Bacterial numbers were measured by Flow cytometry (BD Accuri C6 cytometer) and by counting Colony Forming Units (CFU) after plating on Columbia agar with 5% sheep blood (Biomerieux) flowed by overnight incubation at 37°C.

### 2.3 Cell cultures and infections

To prepare bone marrow-derived dendritic cells, femurs and tibia were harvested from C57BL/6 female mice and bone marrows were flushed. Cell suspensions were filtered through cell strainer 100µm (BD Falcon) and resuspended in RPMI medium supplemented with 10% fetal calf serum (FCS, Gibco), 10mM HEPES (Gibco), 50µM β-mercaptoethanol (Gibco) and 50 µg.ml^-1^ gentamycin (Gibco). Cells were plated at 2.10^6^ cells per ml in medium containing 100ng.ml^-1^ recombinant human Flt3L (Amgen) in order to generate Flt3L-derived DCs (de Brito et al., 2011). Cells were used after a 7-days incubation at 37°C and 7% of CO_2_. The percentage of conventional and plasmacytoid DCs in these cultures are 80% and 20%, respectively (de Brito et al., 2011).

Effector F5 CD8^+^ T cells were generated from F5 transgenic mice. Spleens were harvested and filtered through nylon cell strainer 100µm (BD Falcon). Splenocytes were cultured in DMEM medium supplemented with 10% FCS, 10mM HEPES, 50µM β-mercaptoethanol, 50 µg.ml^-1^ gentamycin, 10% supernatant containing murine IL-2 (obtained from the culture supernatants of the mouse myeloma clone X63-Ag8.653 cell lines transfected with mouse IL-2 gene (Karasuyama and Melchers, 1988)) and 30nM NP68 peptide (Proteogenix, sequence: ASNENMDAM). Alternatively, T cells were sorted from spleens (“CD8a^+^ T Cell Isolation Kit, mouse”, Miltenyi) and cultured with pulsed (NP68 peptide, 20nM) and activated (CpG 1826, 2 µg/ml, Invivogen) Flt3L-derived DCs in the presence of 10% supernatant containing murine IL-2. Cells were cultured at 37°C and 7% CO_2_ for five days.

The DC2.4 cell line (Shen et al., 1997) was cultured in RPMI medium supplemented with 10% FCS, 10mM HEPES, 50µM β-mercaptoethanol and 50 µg.ml^-1^ gentamycin and incubated at 37°C and 5% CO_2_.

DCs were seeded in 96 U-well plates (Dutscher Scientific) at 6.10^4^ (DC2.4) or 1.10^5^ (Flt3L-derived DCs) cells per well in RPMI medium supplemented with 10% FCS, 10mM HEPES and 50µM β-mercaptoethanol but no antibiotics. DCs were incubated with infectious *S. aureus* at the indicated multiplicity of infection (MOI) for 1h, washed with Phosphate Buffer Saline (PBS, Gibco) and further incubated for the indicated time with RPMI medium containing antibiotic (50 µg.ml^-1^ gentamycin, Gibco) in order to kill extracellular *S. aureus*. When indicated, DCs were incubated with equivalent numbers of heat-killed (96°C for 30 min) *S. aureus.*

### 2.4 Antibody staining and cytometry

Flt3L-derived DCs were first labeled with fixable viability dye eFluor 780 (Invitrogen) 20 min at 4°C in PBS. To minimize non-specific binding of antibodies, samples were incubated with Fc block 2.4G2 supernatant in PBS for 10 min. Then samples were antibody-stained for 30 min at 4°C in PBS, 1% FCS (Hyclone) and 0.9% sodium azide. The following antibodies were used: anti-CD11c (PerCP-Cy5, N418 clone, Biolegend), anti-CD80 (BV605, 16-10A1 clone, BD Biosciences), anti-CD86 (PE-Cy7, GL-1 clone, Biolegend), anti-CD40 (BV421, 3.23 clone, BD Biosciences), anti-B220 (APC, RA3-6B2 clone, BD Biosciences) and anti-CMHI H2Db (PE, ER-HR52 clone, Biorad). After washes, samples were fixed for 20 min with Cytofix/Cytoperm buffer (BD Biosciences). Data were acquired on a LSR Fortessa 4 lasers flow cytometer (BD Biosciences). Data analysis was then performed using FlowJo software (Tree star, version 10).

### 2.5 Quantification of cytokines

Culture supernatants were collected and stored at -20°C. Levels of IFN-γ and IL-2 were determined using enzyme-linked immunosorbent assay (ELISA) according to the manufacturer’s recommendations (Biolegend).

### 2.6 Cytotoxic assay

F5 effector cells were stained with CellTrace Violet (Invitrogen, according to the manufacturer’s recommendations) and cultured with *S. aureus* infected DC2.4 at the indicated ratios for 4h. As control, F5 effector cells were incubated with DC2.4 loaded with 20nM of NP68 peptide. Cells were stained with fixable viability dye eFluor 780 (Invitrogen) 20 min at 4°C in PBS and fixed for 20 min with Cytofix/Cytoperm (BD Biosciences). Data were acquired on a LSR Fortessa 4 lasers flow cytometer (BD Biosciences). Data analysis was then performed using FlowJo software (Tree star, version 10).

### 2.7 Confocal microscopy

DC2.4 cells were left to adhere on glass coverslips for 1h before infection with *S. aureus* as described above. Specimens were fixed with paraformaldehyde 2% (Invitrogen) for 10 min, permeabilized with Triton 0.5% (Sigma) for 15 min, before staining with Phalloidin-TRITC (10 µg.ml^-1^, Abcam) for 30 min. EEA-1 was detected using primary antibody (440ng.ml^-1^, F.43.1 clone, Invitrogen) and revealed with secondary antibody anti-rabbit conjugated with AF647 (8 µg.ml^-1^, Invitrogen). Coverslips were then mounted with Prolong Gold antifade reagent (Invitrogen) containing DAPI (1µg/ml, Invitrogen). Images were acquired with a LSM 980 microscope (Zeiss) and analyzed with ImageJ (version 2.1.0). Images displayed are the Mean Intensity Projection of 10 - 20 slices.

### 2.8 Time lapse microscopy

DC2.4 were stained with CellTrace Deep Red (Invitrogen, according to the manufacturer’s recommendations), co-cultured in 96 wells plates with GFP-NP68 *S. aureus* and imaged using CQ1 microscope (Yokogawa). Effector F5 T cells stained with CellTrace Violet (Invitrogen, according to the manufacturer’s recommendations) were added to the cultures. Images displayed are the Mean Intensity Projection from 10 - 20 slices.

### 2.9 Statistics

Statistical analyses (t-tests) were performed using Prism software (GraphPad, version 9). Levels of significance are expressed as p-values (* p<0.05, ** p<0.01, *** p<0.001, ****p<0.0001). The bars represent the means ± SD of replicates or of independent experiments.

## 3 Results

### 3.1 *S. aureus* is internalized by murine bone marrow dendritic cells and induces their maturation

In order to investigate CD8^+^ T cell immune responses against intracellular *S. aureus*, we generated SH1000 *S. aureus* strains that express fluorescent GFP tracer protein and the NP68 epitope. Although it is not as invasive as other *S. aureus* strains, we chose SH1000 strain as it infects many cell types and activates their cytokine production while preserving cell viability (Strobel et al., 2016).

We first wished to establish whether dendritic cells (DCs) can be infected by *S. aureus in vitro*. We used murine bone marrow-derived DCs in the presence of FMS-like tyrosine kinase 3 ligand (Flt3L DCs), these cells representing *bona fide* counterparts of the splenic DC subsets (Naik et al., 2005; Dresch et al., 2012). To follow *S. aureus* infection and its impact on Flt3L DCs, cells were co-cultured with SH1000 GFP-NP68 at a multiplicity of infection (MOI) of 10. DCs were washed and further cultured with gentamycin, at a dose that does not kill intracellular *S. aureus* (Mohamed et al., 2014). The viability of Flt3L DCs along with the expression of GFP among CD11c^+^ DCs were monitored 24, 48- or 72-hours post-infection (pi) by flow cytometry. As control, Flt3L DCs were also incubated with heat-killed *S. aureus*.

**Figure 1:**
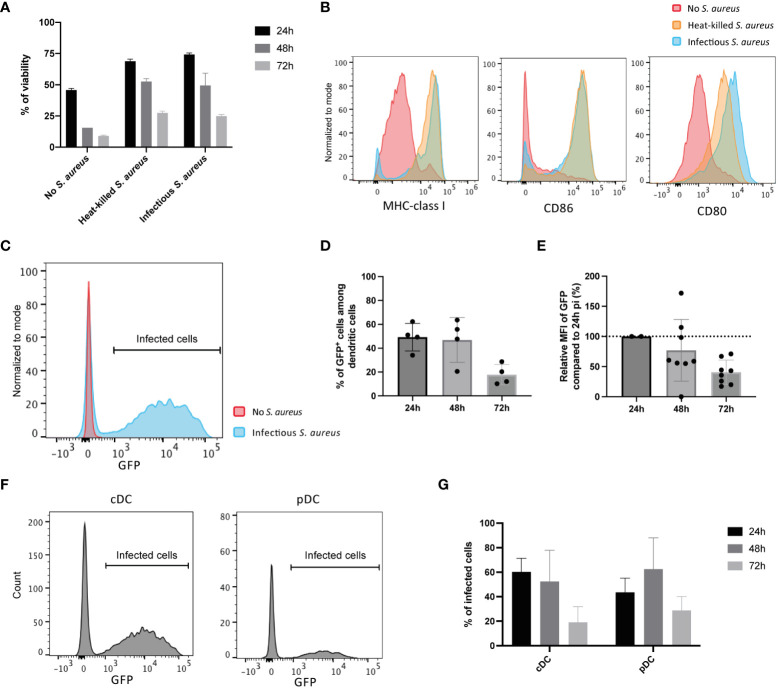
Internalization of *S. aureus* by primary bone marrow-derived dendritic cells. FIt3L DCs were cultured in the absence or in the presence of live infectious *S. aureus* strain SH1000 expressing GFP and NP68 epitope (for 1h), at a MOI of 10, washed and further cultured the indicated time periods. Co-culture with heat-killed *S. aureus* (equivalent of 10 bacteria per DC) was also performed (16h). **(A)** Viability of Flt3L DCs after 24-, 48- or 72- hours (n=4 replicates, results are representative of 2 experiments). **(B)** Cell surface expression of CD80, CD86 and MHC-I at 24h pi assessed by flow cytometry. **(C)** GFP expressing cells among CD11c^+^ DCs at 24h pi. **(D)** Percentages of live CD11c^+^ DCs being GFP positive at 24h, 48h or 72h pi (n=4 replicates, results are representative of 2 independent experiments). **(E)** GFP expression levels relative to the one at 24h pi (n=2 independent experiments). **(F)** Histogram of GFP expression among CD11c^+^ B220^-^ conventional DCs (cDCs) and CD11c^+^ B220^+^ plasmacytoid DCs (pDCs) 24h pi. **(G)** Percentages of infected (GFP) cells among cDCs and pDCs live cells at 24h, 48h or 72h pi (n=3 replicates, results are representative of 2 experiments).

Infection of Flt3L DCs with SH1000 *S. aureus* strain did not induce DCs cell death (**Figure 1A**) and even delayed the decrease in cell viability associated with the spontaneous death of these primary cells. This delay was not due to the infection step as it is also observed when DCs are in contact with heat-killed *S. aureus*. This delay is rather likely related to Flt3L DCs maturation, illustrated by the increase in MHC-I, CD86 and CD80 expression when cells were cultured with infectious or heat killed *S. aureus* (**Figure 1B**). One hour of contact with live infectious *S. aureus* led to 50% of Flt3L DCs containing viable *S. aureus* as measured one day later (**Figure 1C** and data not shown). However, the percentage of infected cells decreased with time as well as the median of fluorescence (MFI) of GFP among cells (**Figures 1C, D**). Flt3L DCs contain two distinct populations, conventional DCs (cDCs, CD11c^+^ B220^-^) and plasmacytoid DCs (pDCs, CD11c^+^ B220^+^), both of them being equally infected by *S. aureus* (**Figures 1F, G**). Taken together, these results show that *S. aureus* is internalized by primary mouse Flt3L DCs and does not seem to proliferate within these cells under our experimental conditions.

### 3.2 Characterization of *S. aureus* internalization by DC2.4 dendritic cell line

**Figure 2:**
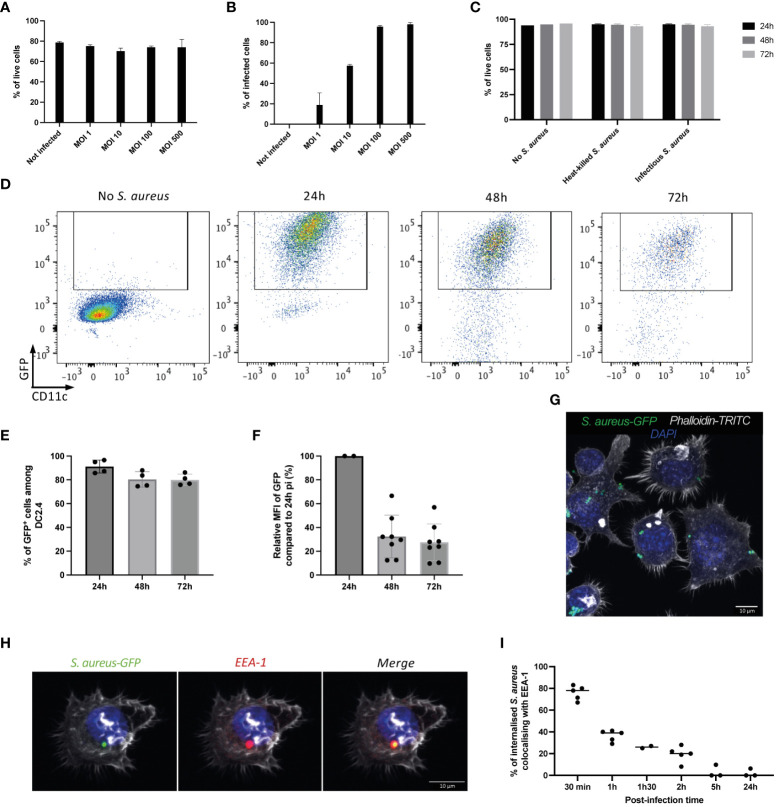
DC2.4 dendritic cells infection by *S. aureus*. DC2.4 were left untreated, incubated for 16h with GFP-NP68 heat-inactivated *S. aureus* or cultured with infectious SH1000 GFP-NP68 expressing strain for 1h, washed and further cultured for 24, 48 or 72h. Increasing concentrations of *S. aureus* (MOI of 1, 10, 100 or 500) were used and viability **(A)** or percentage of GFP cells **(B)** were measured by flow cytometry 24h pi (n=2 replicates, results are representative of 2 independent experiments). **(C)** Viability at 24-, 48- or 72-hours pi (MOI of 10, n=4 replicates, results are representative of 3 experiments), **(D)** GFP and CD11c expression measured by flow cytometry 24h, 48h or 72h pi (MOI of 10). **(E)** Percentages of GFP expression (n=4 replicates, results are representative of 3 experiments). **(F)** GFP expression levels relative to the one at 24h pi (n=4 replicates from 2 experiments). **(G)** Confocal microscopy imaging of DC2.4 cells 1h pi (MOI of 10, nucleus stained with DAPI, actin with phalloidin). **(H)** Confocal microscopy imaging of DC2.4 cells 30 min pi (MOI of 10, nucleus stained with DAPI, actin with phalloidin and early-endosomes with anti-EEA-1 antibody). **(I)** Percentages of co-localization between EEA-1 and GFP were assessed at different time points post-infection (n=2 to 5 microscopic fields from one experiment, representative of 2 independent experiments).

The study was then extended to DC2.4 cell line, an immortalized mouse dendritic cell line that express dendritic cell-specific markers such as DEC-205 and 33D1 and high levels of MHC and co-stimulatory molecules (Shen et al., 1997). As primary DCs, DC2.4 cells have the ability to phagocytose and present exogenous antigens on MHC-I (Shen et al., 1997). DC2.4 were incubated with different MOI of infectious GFP-NP68 expressing SH1000 strain for 1h, washed and further incubated in the presence of gentamycin antibiotic. **Figure 2** shows that *S. aureus* internalization did not affect DC2.4 survival regardless of the MOI (**Figures 2A, C**). The percentage of infection was proportional to the MOI, reaching a maximum at MOI 100 (**Figure 2B**). The viability of infected cells was maintained over a 3 days period (**Figure 2C**), as well as the percentage of GFP-positive DC2.4 (**Figures 2D, E**). The levels of GFP within each DC decreased with time (**Figure 2F**), reflecting the killing of *S. aureus* and/or the proliferation of DC2.4 (**Supplementary Figure 1**). Indeed, shortly after the infection, the majority of DC2.4 cells contained several bacteria (**Figure 2G**; **Supplementary Video 1**) which were distributed between daughter cells during proliferation. Internalized bacteria remained alive and a fraction acquired a SCV phenotype (**Supplementary Figure 1**). Early endosome antigen-1 (EEA-1) is an endosome-specific peripheral membrane protein found in early phagosomes. EEA-1 colocalized with more than 80% of *S. aureus* at 30 min pi indicating that the internalized bacteria trafficked via the endosomal pathway. Endosomal vacuoles matured further as indicated by the loss of EEA-1 staining (**Figures 2H, I**).

### 3.3 Effector CD8^+^ T cells in contact with *S. aureus* infected dendritic cells produce IFN-γ after an antigen-specific recognition

**Figure 3:**
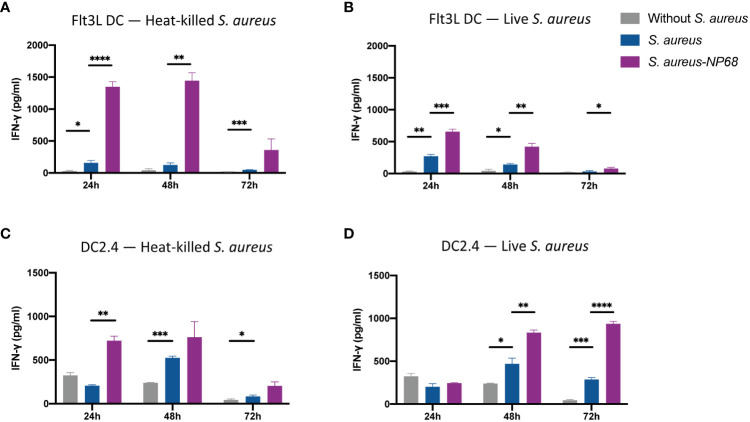
Antigen-specific IFN-γ production by F5 CD8^+^ T cells in response to *S. aureus*-infected dendritic cells. Flt3L DCs **(A, B)** and DC2.4 **(C, D)** were cultured with equivalent numbers of heat-inactivated *S. aureus* **(A, C)** or infectious live *S. aureus* **(B, D)**. DCs were cultured without (grey) or with *S. aureus* strain expressing the NP68 (purple) or not (blue) *S. aureus* strain. 24h, 48h or 72h later, effector NP68-specific F5 CD8^+^ T cells were added. 16h later, supernatants were collected and concentration of IFN-γ was quantified by ELISA. *p<0.05, **p<0.01, ***p<0.001, ****p<0.0001, multiple unpaired t-test (triplicate samples, results are representative of 4 independent experiments).

Having established that Flt3L DCs and DC2.4 can be infected by *S. aureus*, we used these two populations to study the ability of DCs to present *S. aureus-*derived antigens at their surface on MHC-I molecules to CD8^+^ T cells. Dead heat-killed NP68 expressing *S. aureus* was used as a positive control as it was likely that phagocytosed dead bacteria would enter the cross-presentation pathway allowing cross-presentation of *S. aureus*-derived epitopes on MHC-I molecules to CD8^+^ T cells (**Figures 3A, C**). 24-, 48- or 72-hours after DC co-culture with *S. aureus*, effector F5 CD8^+^ T cells recognizing NP68 epitope were added to the cultures. If the TCR of F5 CD8^+^ T recognize their cognate NP68 epitope presented onto MHC-I proteins, F5 CD8^+^ T cells get stimulated, which can be followed by measurement of cytokine production (Brinza et al., 2016). The production of IFN-γ in the supernatant was measured 16h after the addition of F5 effector cells (**Figure 3**). F5 T cells produced more IFN-γ when cultured with Flt3L DCs and DC2.4 infected with control *S. aureus* compared to uninfected DCs. This is also true for the interleukin-2 (IL-2) production (**Supplementary Figure 2**). This activation, which is not antigen specific, likely reflects an activation of effector CD8^+^ T cells interacting with DCs that have matured in contact with *S. aureus* (**Figure 1**; **Supplementary Figure 3**). Nevertheless, the production of IFN-γ was much higher when F5 CD8^+^ T cells interacted with DCs having internalized heat-killed and live *S. aureus* that express NP68. These results demonstrate that the NP68 epitope expressed by *S. aureus* is presented at the surface of DCs and is recognized by F5 CD8^+^ T cells.

The decrease of IFN-γ production from Flt3L DCs cultures with time reflects the death of the primary cells. Interestingly, when DC2.4 were loaded with heat-killed NP68 expressing *S. aureus*, IFN-γ production occurred rapidly and decreased over time, likely reflecting a decrease in antigen cross-presentation due to antigen depletion 72h after engulfment of heat-killed *S. aureus*. In contrast, there was an increase of IFN-γ production by F5 CD8^+^ T cells over time when they were in contact with DC2.4 that had internalized NP68-expressing live *S. aureus*. This likely reflects a continuous synthesis and an accumulation of NP68 antigen by live bacteria within DC2.4 (**Figure 3D**).

### 3.4 Cytotoxicity of effector CD8^+^ T cells against *S. aureus*-infected DC2.4

**Figure 4:**
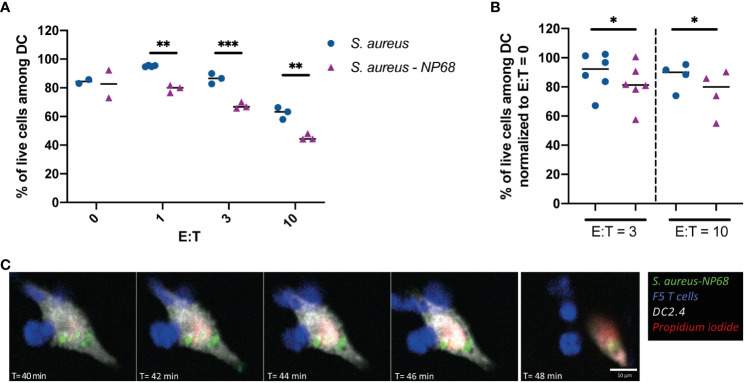
Antigen-dependent F5 CD8^+^ T cell cytotoxicity against *S. aureus*-infected DC2.4. **(A, B)** DC2.4 were cultured with *S. aureus* expressing (purple triangles) or not (blue circles) the NP68 epitope. Effector F5 CD8^+^ T cells were added 24h later at the indicated ratios (E:T) and the viability of DC2.4 was measured by flow cytometry 4h later. **(A)** Results from one experiment with replicates. **(B)** Results from 6 independent experiments (viability was normalized using the percentage of live DCs in the absence of T cells). *p<0.05, **p<0.01, ***p<0.001, multiple unpaired t-test. **(C)** Time-lapse imaging (40 to 48 min pi) of one DC2.4 cell (grey) infected with GFP-NP68 expressing *S. aureus* (green) cultured with effector F5 CD8^+^ T cells (blue). Medium contained propidium iodide (red) that labels dying DCs. One crop of one time-lapse out of two is shown.

One of the main functions of cytotoxic CD8^+^ T cells is the specific killing of infected cells. Therefore, we compared the ability of effector F5 T cells to kill DC2.4 infected by *S. aureus* strain expressing or not the NP68 epitope. It is worth noticing that DC2.4 cells are resistant to CD8^+^ T cell killing compared to other cell types widely used in cytotoxicity assays such as EL4 cells (**Supplementary Figure 4**). However, EL4 cells could not be used as they do not get infected by *S. aureus* (data not shown). At the higher CD8^+^ T cells:DCs ratio, F5 CD8^+^ T cells induced killing of DC2.4 infected with a *S. aureus* strain that did not express NP68. This observation illustrates a non-antigen specific activation of F5 CD8^+^ T cells (**Figure 4A**). However, the cytotoxicity of F5 CD8^+^ T cells toward DC2.4 was significantly increased when *S. aureus* expressed NP68 antigen (**Figures 4A, B**). Altogether these results indicate that F5 CD8^+^ T cells can recognize and kill in an antigen specific manner cells that have internalized *S. aureus* bacteria (illustrated in **Figure 4C**; in **Supplementary Video 1**).

## 4 Discussion

In this study, we evidenced that primary Flt3L-derived DCs and DC2.4 cell line can be efficiently infected by *S. aureus*. Using a *S. aureus* engineered to express a model peptide, we demonstrated that peptides derived from *S. aureus* can get presented at the surface of infected DCs, triggering the activation of antigen-specific effector CD8^+^ T cells as well as the killing of infected DCs by T cells.

To study CD8^+^ T cell immune responses against intracellular *S. aureus*, we used DCs as these cells efficiently recognize a wide range of invading microorganisms and play a critical role in shaping the adaptive immune responses against *S. aureus*. In a mouse model of systemic infection, DCs are rapidly recruited into infected tissues and their depletion using CD11c-DTR transgenic mice results in substantial worsening of infection (Schindler et al., 2012). *In vitro*, only BDCA1^+^ myeloid DCs are able to engulf *S. aureus* and strongly upregulate the expression of costimulatory molecules and production of proinflammatory cytokines, inducing T cell differentiation into IFN-γ-producing CD4^+^ and CD8^+^ T cells (Jin et al., 2014). The capacity of DCs to internalize *S. aureus* and their ensuing maturation is consistent with previous results obtained *in vitro* (Balraadjsing et al., 2019) or *in vivo* (Zhang et al., 2020). This maturation could partly be due to the activation of the TLR2-MyD88 pathway after the recognition of *S. aureus* lipoproteins as shown *in vivo* (Schmaler et al., 2011; Darisipudi et al., 2018). In our study we observed that culture of DCs with *S. aureus* SH1000 strain leads to bacteria internalization and DC maturation. In the experimental conditions we have used, the bacteria that have been internalized remained viable and formed a reservoir with a small proportion of bacteria that acquired a SCV phenotype (Löffler et al., 2014).

We showed that F5 CD8^+^ T cells are activated by DCs infected with a *S. aureus* strain expressing their cognate peptide NP68, revealing that the protein harboring the NP68 peptide was expressed within infected cells, processed by the proteasome and exposed at the cell surface on MHC-I molecules. As shown in **Figure 3** there was an increase of IFN-γ production by F5 CD8^+^ T cells over time when they are in contact with DC2.4 that have internalized NP68-expressing *S. aureus*. This likely reflects a continuous synthesis of NP68 antigen that accumulates within DC2.4. IFN-γ has a dual role in the healing of *S. aureus* infection subsequent to the infection stage (Zhao and Tarkowski, 1995). IFN-γ^-/-^ mice recruit less neutrophils upon a surgical wound infection site, resulting in a more efficient clearance of the infecting bacteria (McLoughlin et al., 2008). Nevertheless, others studies indicate a role of IFN-γ for a better clearance of infection in a murine model of infection (Sasaki et al., 2006) or *in vitro* (DeForge et al., 2000; Smith et al., 2010).

The recognition of NP68 epitope from infectious *S. aureus* also leads to an increased killing of infected cells by CD8^+^ T cells. This could participate to the elimination of the bacteria. Indeed, at least in human CD8^+^ T cells, cytotoxic granules contain granulysin which delivers granzymes into bacteria, resulting in the killing of several bacterial strains such as *Listeria monocytogenes* (Walch et al., 2014).

We observed that CD8^+^ T cells were not only activated through the recognition of their specific peptide at the surface of infected DCs but also in the absence of this peptide, albeit at lower levels. This is consistent with studies highlighting the role of superantigens, such as TSST-1, in the activation of T cells (Darisipudi et al., 2018; Balraadjsing et al., 2019; Zhang et al., 2020). Such an activation can also induce the anergy of T cells (O’Hehir and Lamb, 1990; Cornwell and Rogers, 2010), and therefore a failure of T cell response. Antigen-independent production of IFN-γ could also rely on IL-12 and IL-18 production, into the extent that these cytokines have the ability to activate a production of IFN-γ by activated effector cells as shown for memory bystander CD8^+^ T cells (Lee et al., 2022). Heat-killed *S. aureus* have been shown to induce IFN-γ production by resident memory OT-I CD8^+^ T cells *in vivo* (Ge et al., 2019). IL-15 also triggers also memory bystander CD8^+^ T cell activation and induces their upregulation of cytotoxic molecules and NK-activating receptors (such as NKG2D). This upregulation leads to cytotoxic killing activity of bystander-activated CD8^+^ T cells after recognition of stress-induced self-proteins (Lee et al., 2022).

Our results indicate that CD8^+^ T cells have the ability to detect intracellular *S. aureus.* This paves the way to the development of immunotherapies against *S. aureus* chronic infections relying on CD8^+^ T cell responses.

## Data availability statement

The raw data supporting the conclusions of this article will be made available by the authors, without undue reservation.

## Ethics statement

The animal study was approved by CECCAPP, Ministère de l’Enseignement Supérieur et de la Recherche: C2EA15. The study was conducted in accordance with the local legislation and institutional requirements.

## Author contributions

AF planned and performed most experiments, analyzed the data and wrote the manuscript. SD performed some experiments and reviewed the manuscript. SV and PP carried out the design of experiments. MD performed cytokine assays. JB and FV engineered modified *S. aureus*. FV interpreted results and contributed to the writing of the manuscript. JM and YL designed experiments, interpreted results, discussed the results and wrote the manuscript. All authors contributed to the article and approved the submitted version.
