# Supplementary figures and images for: Antigen specific activation of cytotoxic CD8+ T cells by Staphylococcus aureus infected dendritic cells
Source: Front Cell Infect Microbiol. 2023 Oct 26;13:1245299. doi: 10.3389/fcimb.2023.1245299 (PMC10639145; doi:10.3389/fcimb.2023.1245299)

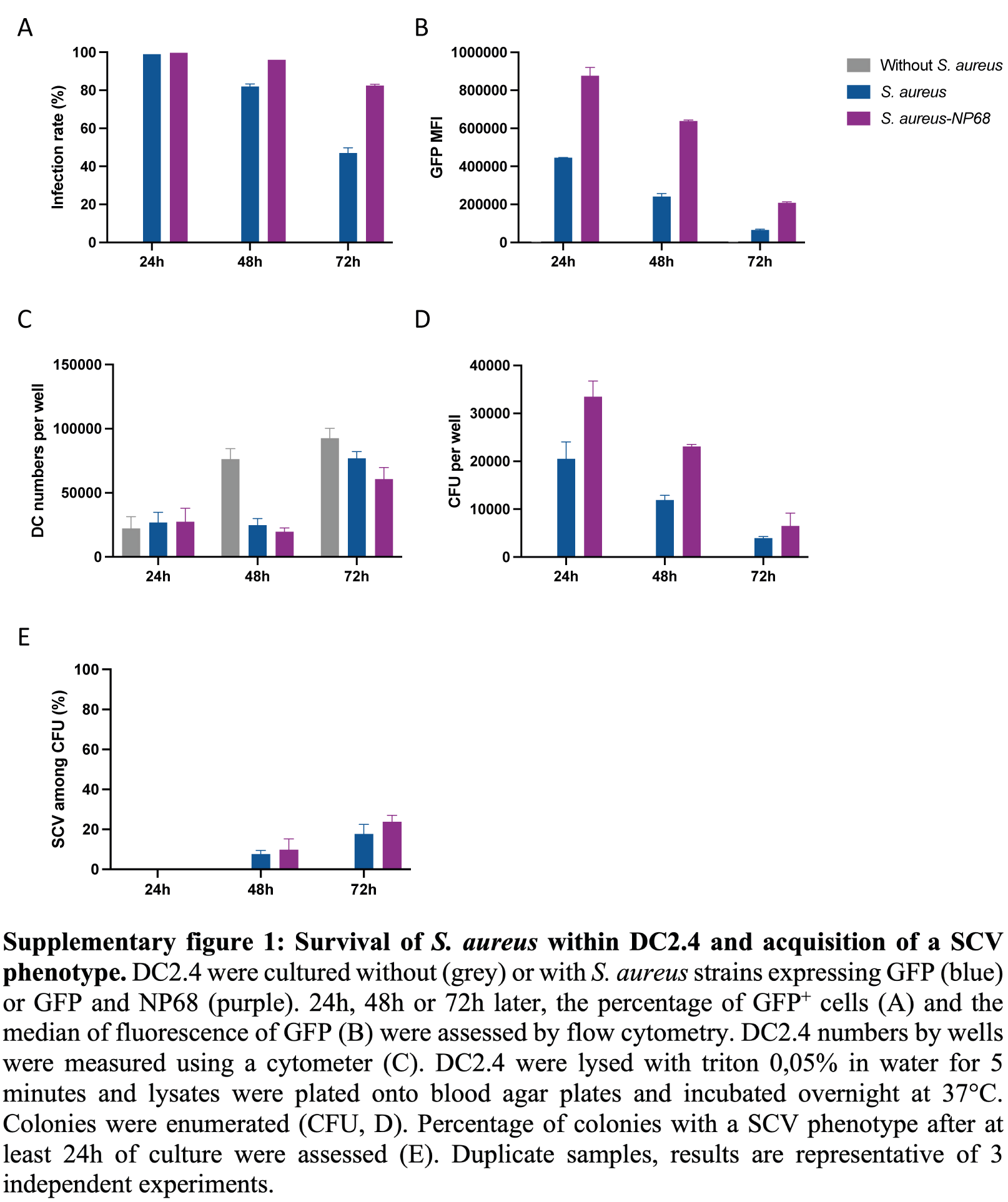

Supplement: Supplementary file 1 [file Image_1.tif]

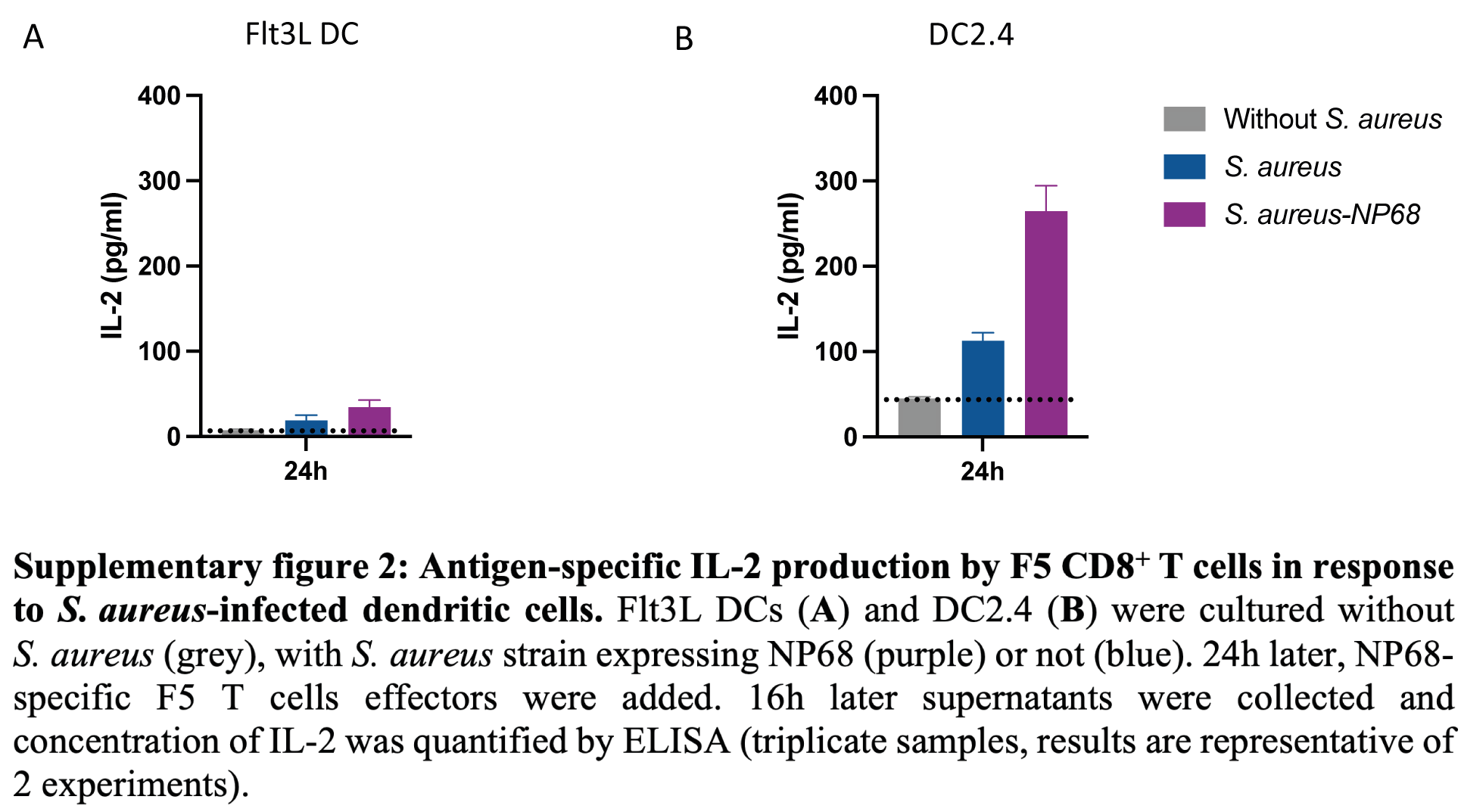

Supplement: Supplementary file 2 [file Image_2.tif]

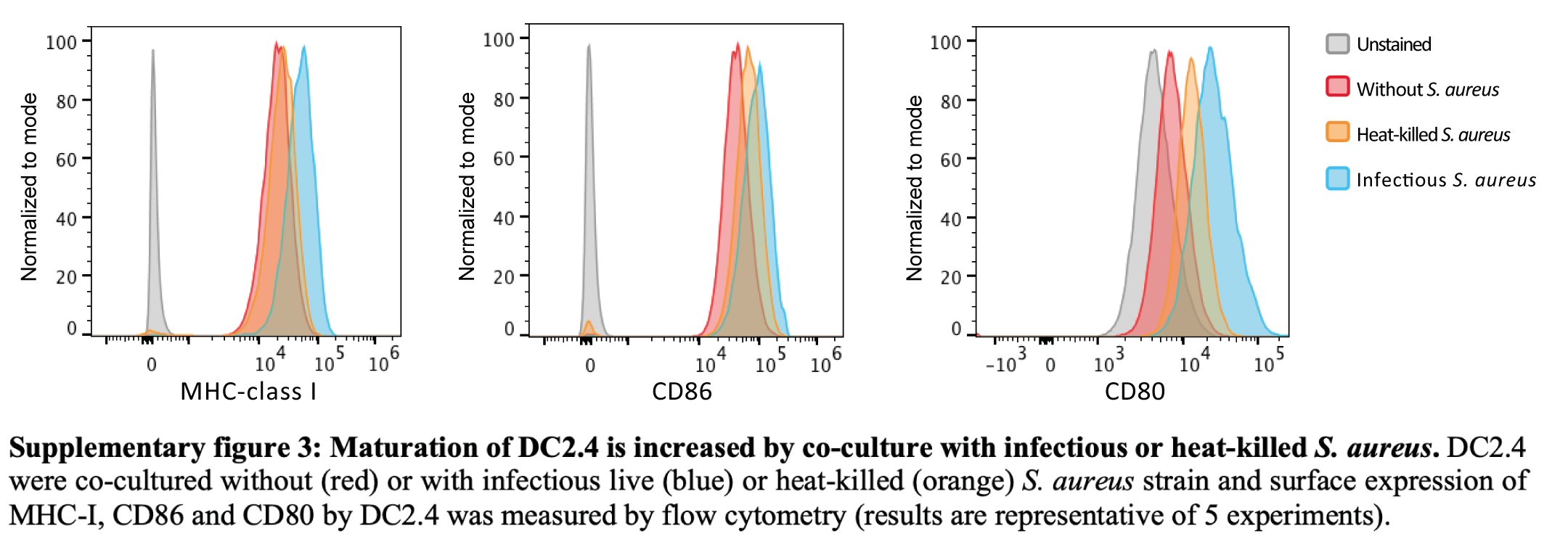

Supplement: Supplementary file 3 [file Image_3.tif]

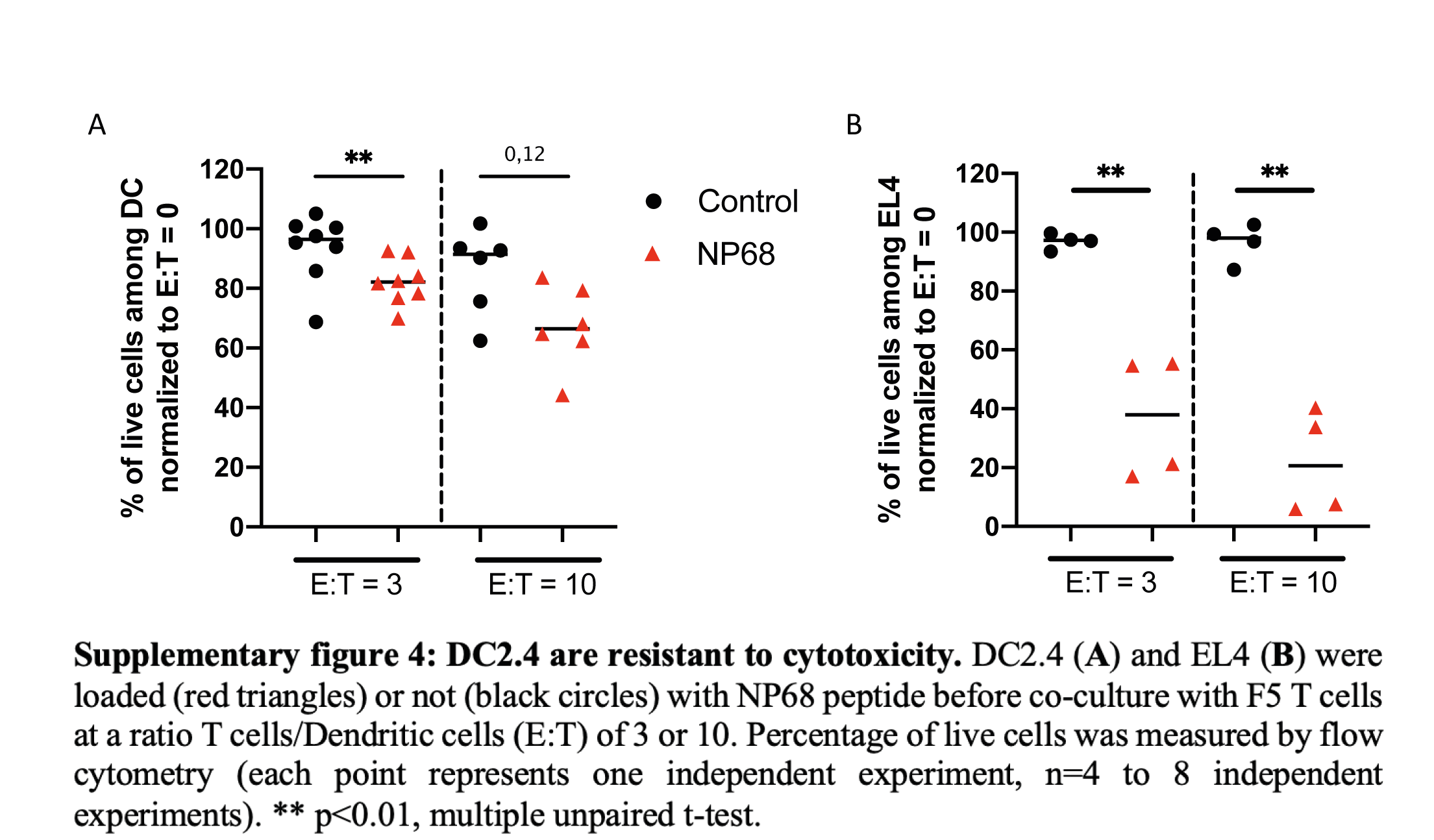

Supplement: Supplementary file 4 [file Image_4.tif]

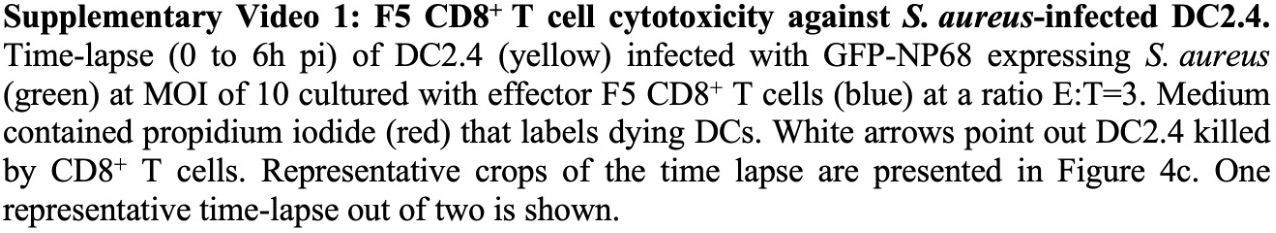

Supplement: Supplementary file 5 [file Image_5.jpg]
